# Supplementary material for: Analysis and design of disordered polypeptides with optimized sequence patterning properties
Source: PLoS Comput Biol. 2026 Jul 8;22(7):e1014462. doi: 10.1371/journal.pcbi.1014462 (PMC13367912; doi:10.1371/journal.pcbi.1014462)
Supplement: S1 Appendix — Component captions are listed below. Fig A. Correlation of different predictive parameters with ensemble-calculated mean and standard deviation of SCD. Fig B. Correlations of the patterning terms SCD, SHD, and SAD for 105 shuffles of the IDPs FUS LC, LAF-1 RGG, and NUP-153. Fig C. FUS LC Monte Carlo runs with no mutations. Multiple parameters are tracked but the trajectories only optimize for targets of the maximum (blue) and minimum (green) possible SCD values. Fig D. Energy decomposition of five parameters with respect to mutations, swaps, and shuffles separately. These also include unaccepted Monte Carlo moves. The range of values observed for the three patterning parameters are within an a few-fold difference for each move type, indicating success in the normalization scheme for application to MC sequence design. Fig E. Per-system correlations between critical temperature and sequence patterning metrics for the designed variants. (A) LAF-1 RGG variants Tc plotted against SCD, SHD, SAD, and the predicted ΔG. Pearson correlation coefficients are indicated in each panel. (B) FUS LC variants. (C) The three mini-NUP designs. Fig F. Approximate 2D phase-behavior map for fixed-composition LAF-1 RGG designs. All 18 LAF-1 RGG variants (WT plus V1–V3 from Table 1 and the 15 additional designs in Table D) are placed at their (SCD,SHD) coordinates and annotated by their simulated critical temperature. Fig G. LAF-1 RGG variant with attenuated phase separation. Coexistence curves obtained from slab simulations for LAF-1 RGG WT (Tc=282.6K) and protein14 (Table D; Tc=274.4K), a fixed-composition variant designed by the same Monte Carlo procedure. Fig H. Multilinear regression of critical temperature on patterning features for LAF-1 RGG designs. (A) Predicted versus observed Tc across the 18 LAF-1 RGG variants from a multilinear regression on the z-score standardized features. (B) Relative feature importance derived from the standardized coefficients. (C) Tc versus SHD. (D) Tc v [file pcbi.1014462.s001.pdf]

# Supporting Information for: Analysis and design of disordered polypeptides with optimized sequence patterning properties

Arjun Singh, Ali I. Ukperaj, Gabriel F. Porto and Gregory L. Dignon

2/17/2026

## SI Figures

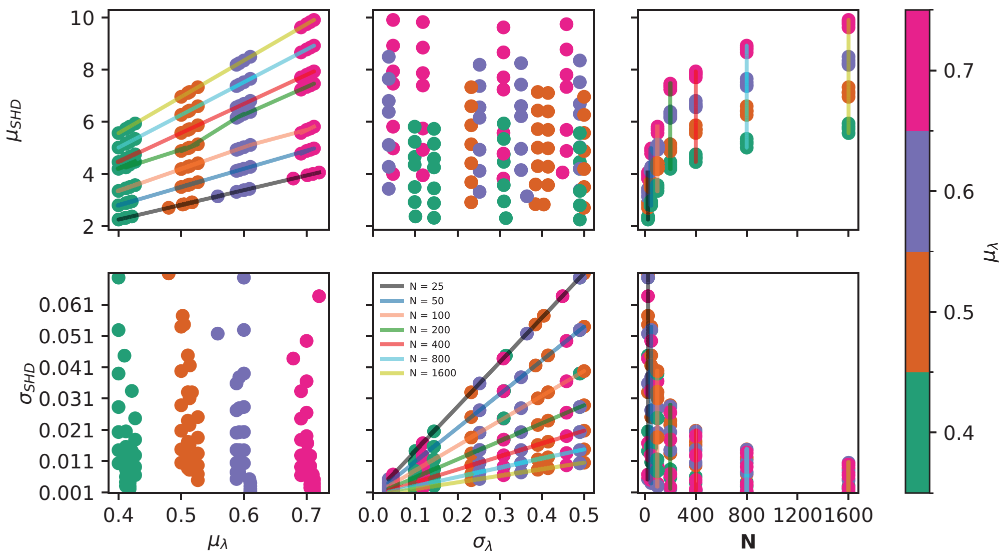


**Fig A.** Correlation of different predictive parameters with ensemble-calculated mean and standard deviation of SHD. Lines connect points of the same length to show trends, they do not represent empirical fits.

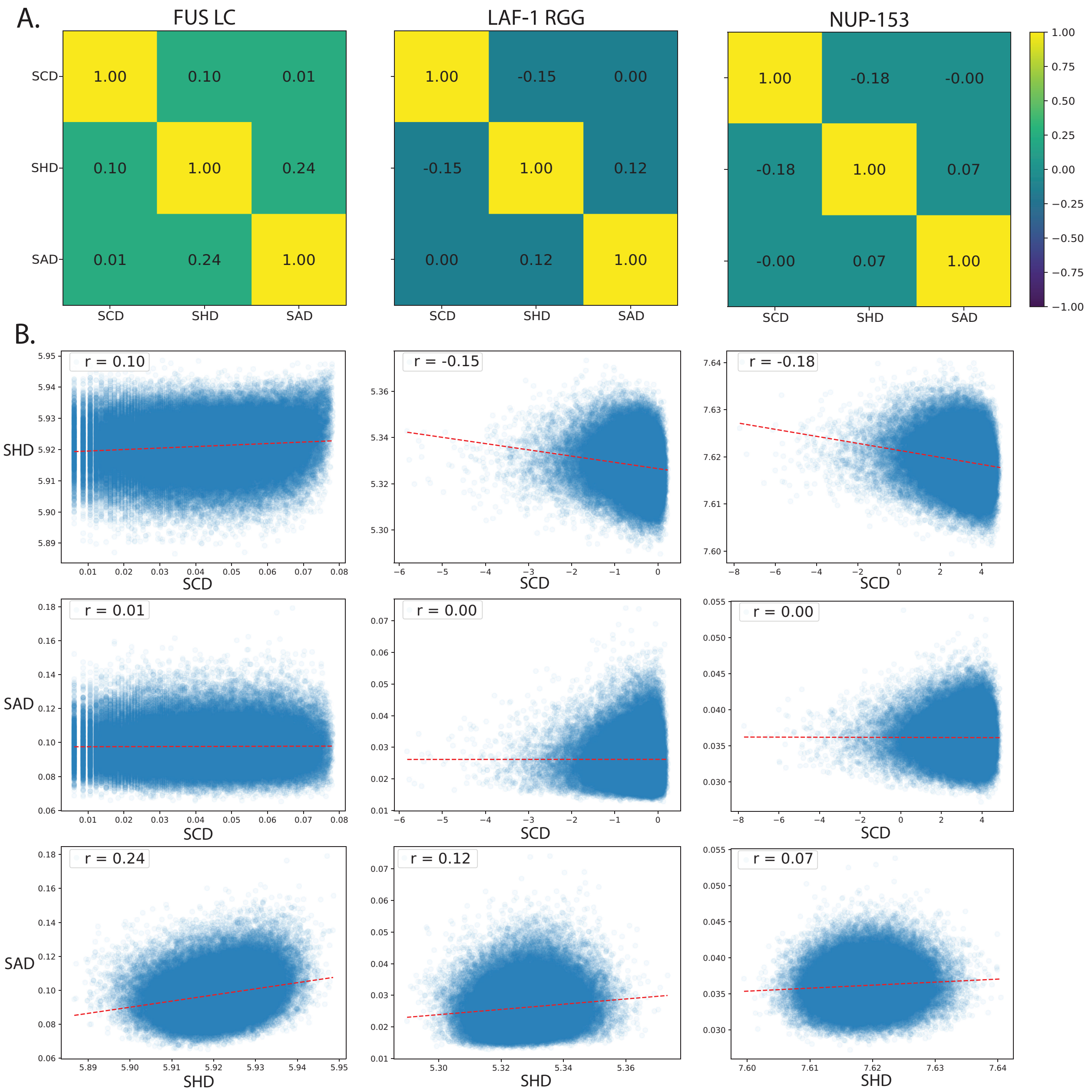


**Fig B.** Correlations of the patterning terms SCD, SHD, and SAD for $10^5$ shuffles of the IDPs FUS LC, LAF-1 RGG, and NUP-153.

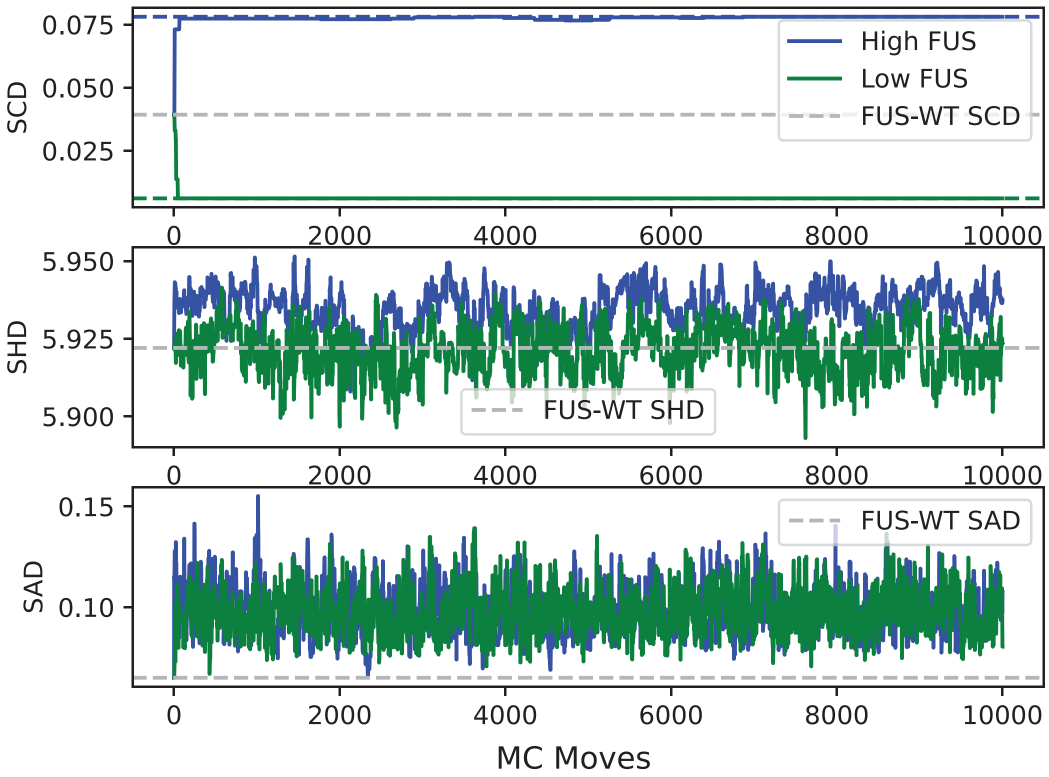


**Fig C.** FUS LC Monte Carlo runs with no mutations. Multiple parameters are tracked but the trajectories only optimize for targets of the maximum (blue) and minimum (green) possible SCD values.

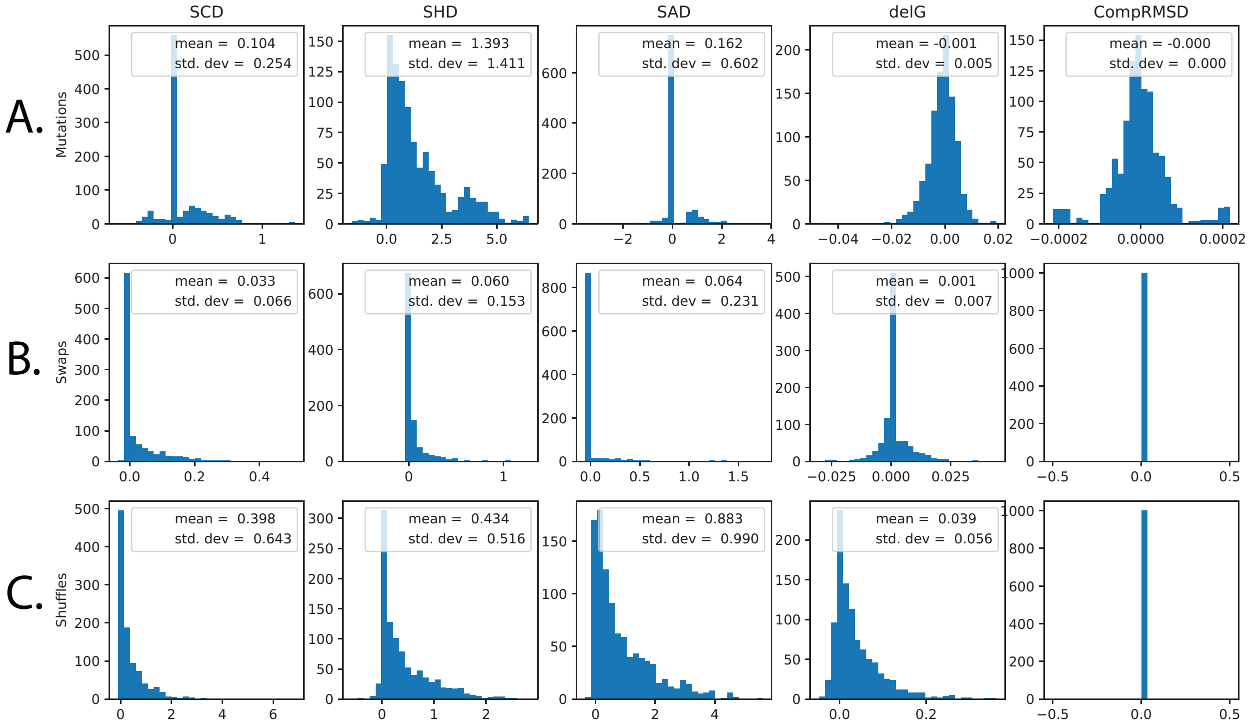


**Fig D.** Energy decomposition of five parameters with respect to mutations, swaps, and shuffles separately. These also include unaccepted Monte Carlo moves. The range of values observed for the three patterning parameters are within an a few-fold difference for each move type, indicating success in the normalization scheme for application to MC sequence design.

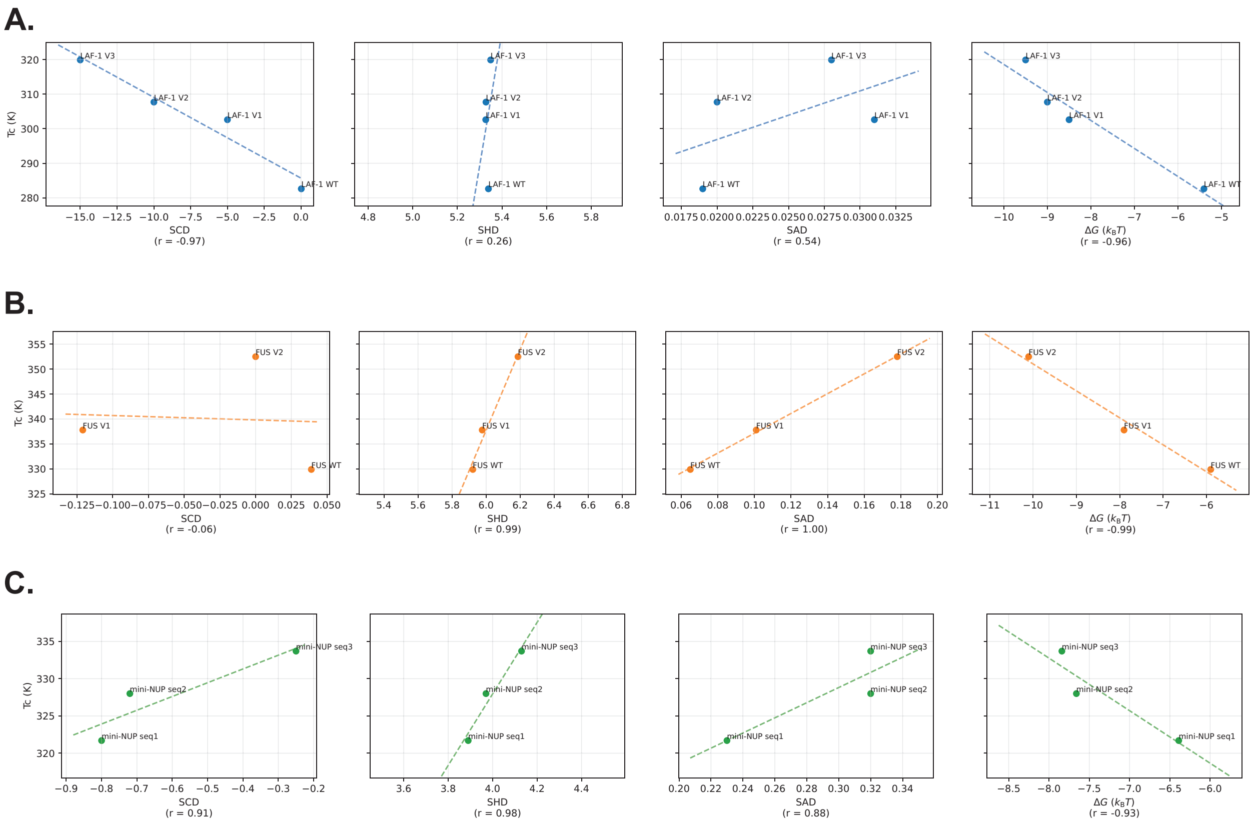


**Fig E.** Per-system correlations between critical temperature and sequence patterning metrics for the designed variants. (A) LAF-1 RGG variants $T_c$ plotted against SCD, SHD, SAD, and the predicted $\Delta G$. Pearson correlation coefficients are indicated in each panel. (B) FUS LC variants. (C) The three mini-NUP designs.

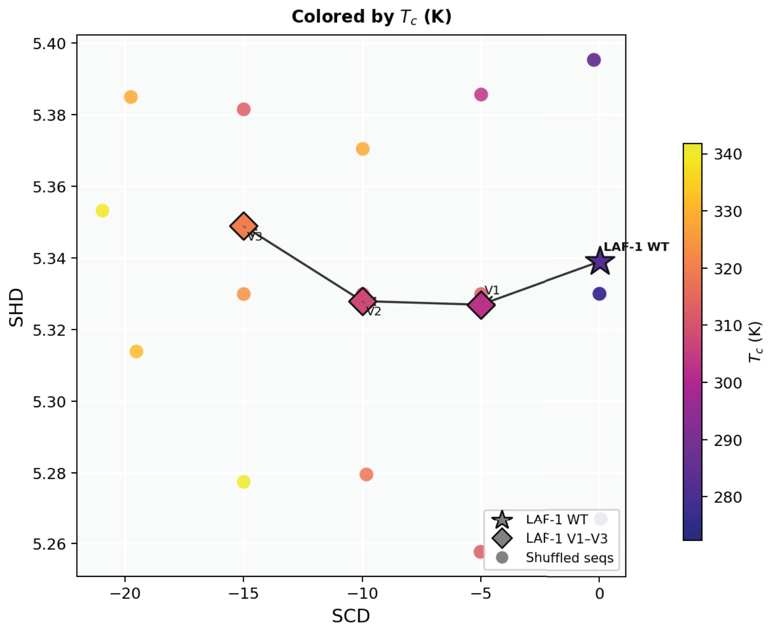


**Fig F.** Approximate 2D phase-behavior map for fixed-composition LAF-1 RGG designs. All 18 LAF-1 RGG variants (WT plus V1–V3 from Table 1 and the 15 additional designs in Table S4) are placed at their $(\mathrm{SCD}, \mathrm{SHD})$ coordinates and annotated by their simulated critical temperature.

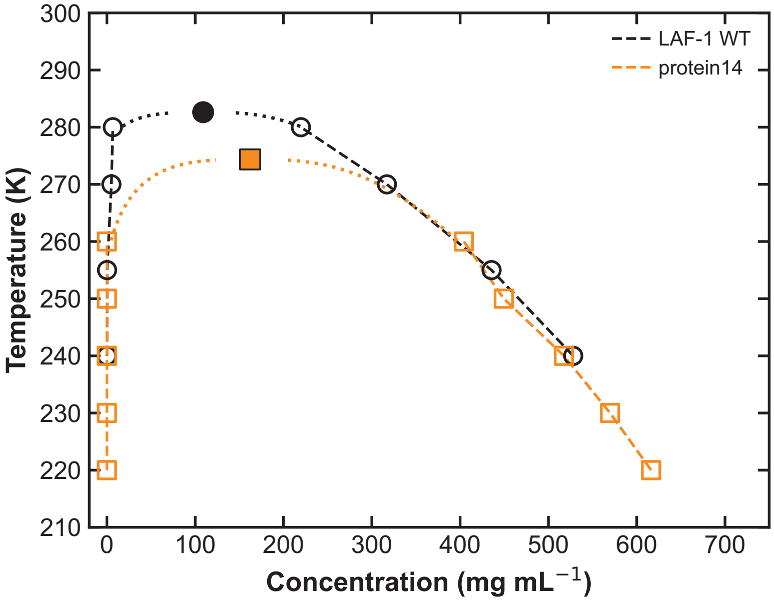


**Fig G.** LAF-1 RGG variant with attenuated phase separation. Coexistence (binodal) curves obtained from slab simulations for LAF-1 RGG WT ($T_c$ = 282.6 K) and `protein14` (Table S4; $T_c$ = 274.4 K), a fixed-composition variant designed by the same Monte Carlo procedure.

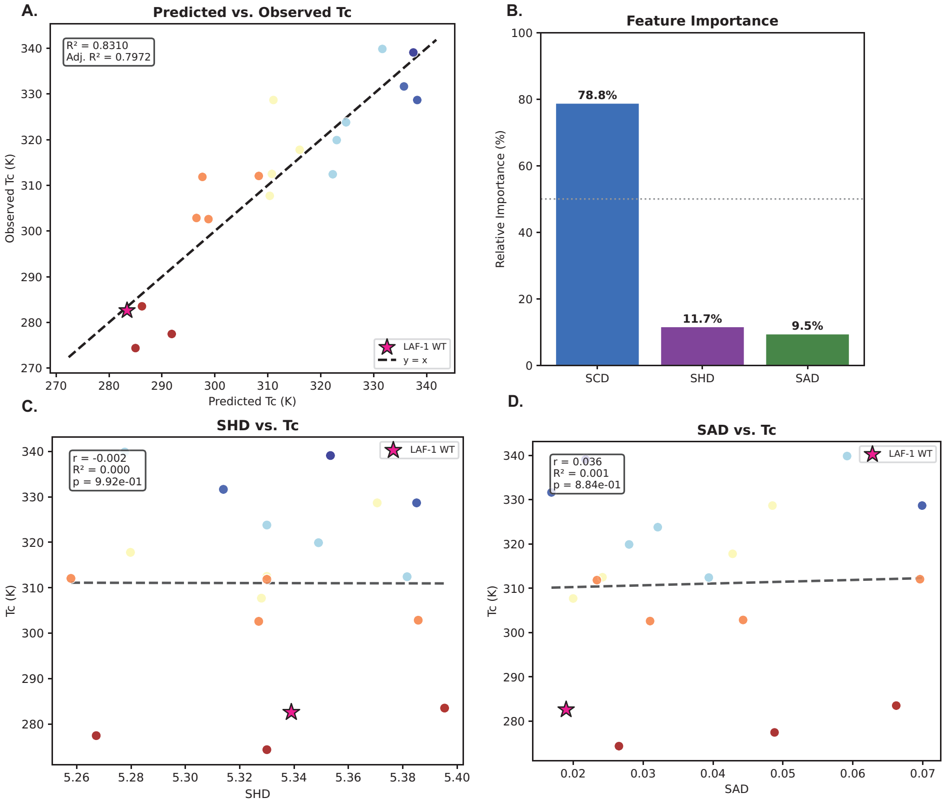


**Fig H.** Multilinear regression of critical temperature on patterning features for LAF-1 RGG designs. (A) Predicted versus observed $T_c$ across the 18 LAF-1 RGG variants from a multilinear regression on the $z$-score standardized features. (B) Relative feature importance derived from the standardized coefficients. (C) Tc versus SHD. (D) $T_c$ versus SAD.

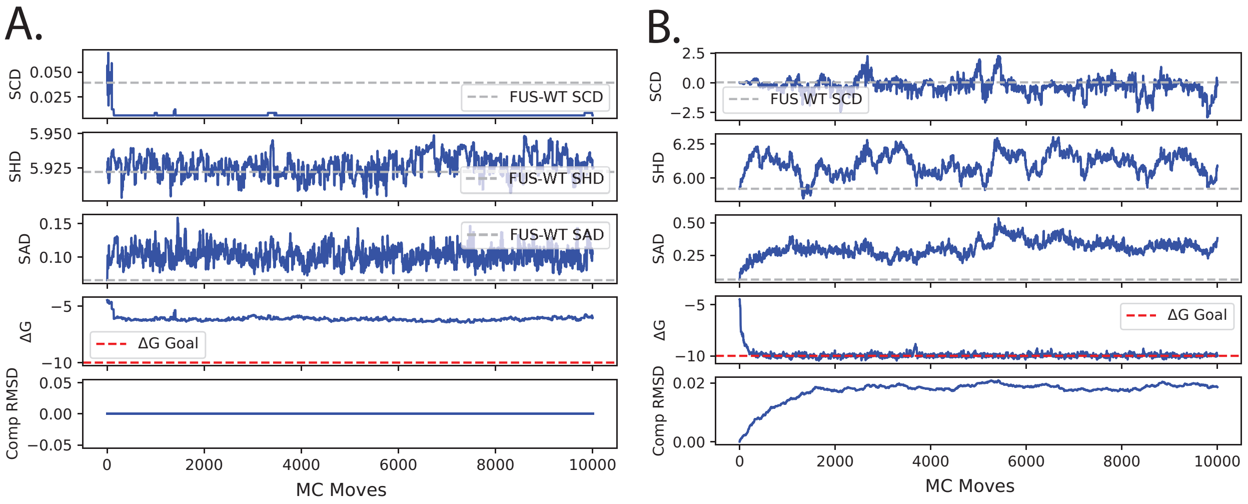


**Fig I.** FUS LC Monte Carlo runs optimizing for only ΔG with (A) no mutations and (B) with mutations enabled.

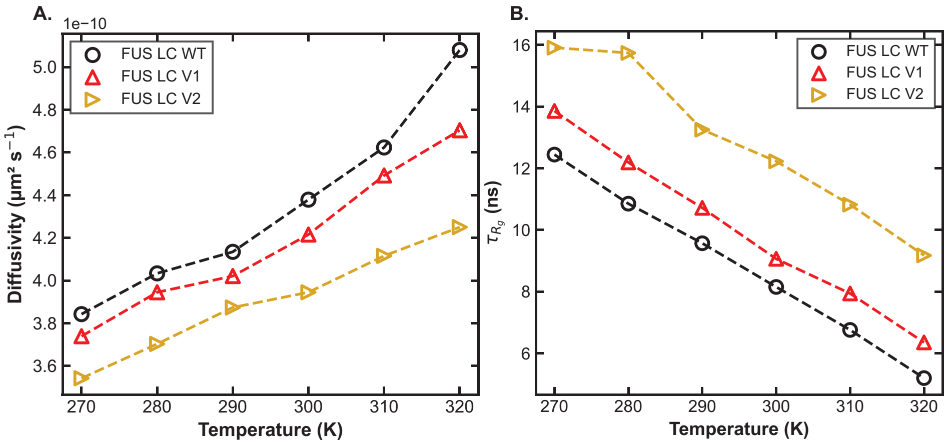


**Fig J.** Temperature dependence of intra-condensate dynamics for FUS LC variants. (A) Single-chain translational diffusivity in the dense phase versus temperature for FUS LC WT, V1, and V2. (B) $R_g$ autocorrelation time $\tau_{R_g}$ versus temperature for the same three sequences.

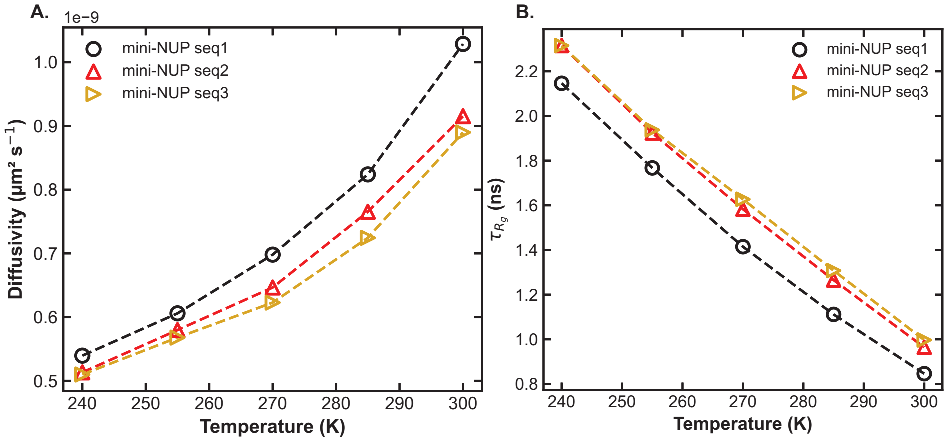


**Fig K.** Temperature dependence of intra-condensate dynamics for the mini-NUP designs. (A) Single-chain translational diffusivity within the dense phase versus temperature for mini-NUP `seq1`, `seq2`, and `seq3`. (B) $R_g$ autocorrelation time $\tau_{R_g}$ versus temperature for the same three sequences.

# SI Tables

| Amino acid | Urry [1] normalized hydropathy scale ($\lambda$) |
|---|---|
| TRP | 1.000000 |
| TYR | 0.897059 |
| PHE | 0.823530 |
| HIS | 0.764707 |
| PRO | 0.758824 |
| LEU | 0.720589 |
| ILE | 0.705883 |
| MET | 0.676471 |
| VAL | 0.664707 |
| CYS | 0.647060 |
| ALA | 0.602942 |
| ASN | 0.588236 |
| SER | 0.588236 |
| THR | 0.588236 |
| GLY | 0.573530 |
| ARG | 0.558824 |
| GLN | 0.558824 |
| LYS | 0.382354 |
| ASP | 0.294119 |
| GLU | 0.000000 |

**Table A.** Normalized hydropathy scales used for the HPS-Urry models.

**Table B.** List of amino acid sequences for real IDPs used in initial shuffling study.

| Protein Name | Length | Sequence |
|---|---|---|
| **LAF-1 IDR WT** | 168 | MESNQSNNGGSGNAALNRGGRYVPPHLRGGDGGAAAAASAGGD DRRGGAGGGGYRRGGGNSGGGGGGGYDRGYNDNRDDRDNRGG SGGYGRDRNYEDRGYNGGGGGGGNRGYNNNRGGGGGGYNRQD RGDGGSSNFSRGGYNNRDEGSDNRGSGRSYNNDRRDNGGDG |
| **FUS WT** | 163 | MASNDYTQQATQSYGAYPTQPGQGYSQQSSQPYGQQSYSGYSQS TDTSGYGQSSYSSYGQSQNTGYGTQSTPQGYGSTGGYGSSQSSQSS YGQQSSYPGYGQQPAPSSTSGSYGSSSQSSSYGQPQSGSYSQQPSYG GQQQSYGQQQSYNPPQGYGQQNQYNS |
| **Nup153** | 1475 | MASGAGGVGGGGGGKIRTRRCHQGPIKPYQQGRQQHQGILSRVT ESVKNIVPGWLQRYFNKNEDVCSCSTDTSEVPRWPENKEDHLVYA DEESSNITDGRITPEPAVSNTEEPSTTSTASNYPDVLTRPSLHRSHL NFSMLESPALHCQPSTSSAFPIGSSGFSLVKEIKDSTSQHDDDNISTT SGFSSRASDKDITVSKNTSLPPLWSPEAERSHSLSQHTATSSKKPAF NLSAFGTLSPSLGNSSILKTSQLGDSPFYPGKTTYGGAAAAVRQSK LRNTPYQAPVRRQMKAKQLSAQSYGVTSSTARRILQSLEKMSSPLA DAKRIPSIVSSPLNSPLDRSGIDITDFQAKREKVDSQYPPVQRLMTP KPVSIATNRSVYFKPSLTPSGEFRKTNQRIDNKCSTGYEKNMTPGQ NREQRESGFSYPNFSLPAANGLSSGVGGGGGKMRRERTRFVASKP LEEEEMEVPVLPKISLPITSSSLPTFNFSSPEITTSSPSPINSSQALTNK VQMTSPSSTGSPMFKFSSPIVKSTEANVLPPSSIGFTFSVPVAKTAE LSGSSSTLEPIISSSAHHVTTVNSTNCKKTPPEDCEGPFRPAEILKEG SVLDILKSPGFASPKIDSVAAQPTATSPVVYTRPAISSFSSSGIGFGE SLKAGSSWQCDTCLLQNKVTDNKCIACQAAKLSPRDTAKQTGIET PNKSGKTTLSASGTGFGDKFKPVIGTWDCDTCLVQNKPEAIKCVA CETPKPGTCVKRALTLTVVSESAETMTASSSSCTVTTGTLGFGDK FKRPIGSWECSVCCVSNNAEDNKCVSCMSEKPGSSVPASSSSTVPV SLPSGGSLGLEKFKKPEGSWDCELCLVQNKADSTKCLACESAKPG TKSGFKGFDTSSSSSNSAASSSFKFGVSSSSSGPSQTLTSTGNFKFGD QGGFKIGVSSDSGSINPMSEGFKFSKPIGDFKFGVSSESKPEEVKKD SKNDNFKFGLSSGLSNPVSLTPFQFGVSNLGQEEKKEELPKSSSAGF SFGTGVINSTPAPANTIVTSENKSSFNLGTIETKSASVAPFTCKTSE AKKEEMPATKGGFSFGNVEPASLPSASVFVLGRTEEKQQEPVTST SLVFGKKADNEEPKCQPVFSFGNSEQTKDENSSKSTFSFSMTKPSE KESEQPAKATFAFGAQTSTTADQGAAKPVFSFLNNSSSSSSTPATS AGGGIFGSSTSSSNPPVATFVFGQSSNPVSSSAFGNTAESSTSQSLLF SQDSKLATTSSTGTAVTPFVFGPGASSNNTTTSGFGFGATTTSSSA GSSFVFGTGPSAPSASPAFGANQTPTFGQSQGASQPNPPGFGSISSS TALFPTGSQPAPPTFGTVSSSSQPPVFGQQPSQSAFGSGTTPNSSSA FQFGSSTTNFNFTNNSPSGVFTFGANSSTPAASAQPSGSGGFPFNQ SPAAFTVGSNGKNVFSSSGTSFSGRKIKTAVRRRK |

**Table C.** List of designed sequences used in this work in MD simulations of phase separation.

| Protein Name | Length | Sequence |
|---|---|---|
| **LAF-1 V1** | 168 | QRNRGYNRGNNGNGGVYGGRSRGRGGALRRGGYYPGSYNSNNR<br>RRGSSGRNRGNRGSGGGRNSAGSDQAGGNGGAANYRRDMSHND<br>YGDGYNARPGESGLGRRGNDDAANNDGYGGGGDGDNGGERGD<br>GGDAGSGGGGYFGGSNEYDGDDGRDGGNNDDGGGGRNGRG |
| **LAF-1 V2** | 168 | RGYYSGNRGRRNRGFSGRGNRLNRYRRNGSRGRPGGLRRGRPSY<br>GRRGGEGGYVRANGGRNNSRGNGSGGGGQNGYAAGGGGGRDY<br>DRGQGGADGYGSSGMGDHAGAGDGNNGGYNNNDAGGNNNGND<br>GRYEGNGANADDEDDGGSRSGGGDDSYNDSDDGGNDGGGG |
| **LAF-1 V3** | 168 | AGADDDNDGELDGDGGDDGNGDNGNDYGDNGEDGDDYYGYPG<br>GGEYRNGGNGGDSNGNSSDNGGNYAGGGNGQDGGNAGGGGRNR<br>GLRFRNGGNYGGGGAGGGSPARGVGYSGNYRNGRRYAANNRSG<br>SGYGGGNSSMGNRRSRRRGRRSHQGRSRRRRRGGGARNGG |
| **FUS V1** | 163 | LASQNQTPADSGYTQPQYPATYQQQGSYSNPQYSGDQSSQQYQYP<br>SGTSYGQSSYGSYGSTSNYGYGGQSTPSGYGSSGGYGHSQSSQQSY<br>GQGSQYSPYGQQLAYGSTGSSSSSGSQGQYSYSGGYKSQTMQPST<br>GIQYQQSYYQQSGQCYGSPQQPNQYNS |
| **FUS V2** | 163 | QQSTTTDPFAQQASQYQQNSGGWTQSYSQSPYSYQQQQWTSSYG<br>GYCSTYQSGQSWQSYGSGYQGYSGFLSGGWGGYQSGYGSSGSWS<br>GGYSIYGGPYSGYSPYNSGSMYYPSSYQYSGPYGSGPQYSQQYQPS<br>QYQQQQYQSNYTSSSSYSTQSQQPQQPQS |
| **mini-NUP 153 seq1** | 30 | NFSKRFPTYQGYIYLGSWSYSFLTFEDEFA |
| **mini-NUP 153 seq2** | 30 | PTFSFRKFGIYSYGYLLYSWNWSFDEFEQA |
| **mini-NUP 153 seq3** | 30 | VAFQFRNFSYMWLGYGYTLIYWDESFSFTP |

**Table D.** List of 15 additional designed LAF-1 RGG variants generated to span the (SCD, SHD) design space at fixed amino acid composition.

| Protein Name | Sequence | $T_c$ (K) | $\rho_c$ | $\Delta G$ |
|---|---|---|---|---|
| **protein1** | RRYARGRRRRNRRGRRGLRGRRGSRRGHARGPGRARRRGNYYR<br>GGNGGRGGGGGNNGGQGYGGSPGGNGNGYGGNYSGGGVRNGG<br>GGYNGGGSGNGDSYGGNNSGSSNGAANNANGSNGMAANGGQYN<br>GGDFDGDDYDLDGNNGGDDDGDDGEADYDDGEDSNGEDSS | 339.08 | 179.61 | -8.4560 |
| **protein2** | RGGGGQGNGGGGGSGNGGGRRGRGGNRQSRRRGRRRRRNGRR<br>RRRRRAGRGLGRNNRSNSRGVGGGNPHNGYGGFGYAYNNNNAM<br>LSGDYYGYNYYGYNSAYPGASGNGGGSGAGYGANGSGDGNDNG<br>NASNNASSNGGGDGDDEDGGGDGDGGDDDGGDGDGDDDEE | 328.67 | 187.80 | -8.4805 |
| **protein3** | RYAYLNRNSRRRFRRRRYRRRNGRRSGNHANRRARRRRGNGGG<br>RPGGGAGGGGGRGRGQGSPGRGSNGYANGGGGNAGGGSGGGN<br>GYSGGGGGGGGSNGQNGGYNGMGGAGGNYDEGSNDDGGGDGN<br>ADDGSYDDGDDDDYDSDDSEDGENSNNYANDGNYNGLNVGG | 331.64 | 184.47 | -8.4250 |
| **protein4** | AYYAYYFSYNNNAYADYNNVASNGDNGGSDGSNNGDDDEGADG<br>DDGDDDSGDGEDGSGGGRGNGGGGSGDGEGDGDGGGNGGGQG<br>RGGGGGGGGGGGNRGGQGNYGGGGGGGSGNNRGGNNSNSRG<br>GGNRLRNRGRRRSRRRRRRRPRRSRNYNPRRANLYAAMYRH | 339.85 | 184.43 | -9.1627 |
| **protein5** | RGSNANRGNNRRVRARRGSRRRGARRRRRNGGRGPGYGRNPFR<br>GGSGGNRMNHRGRGNDRSGGGGGNGYQGGNSGRRGNGNAGDA<br>NEGSQGGNGGSSGGGSYGNGYNAYNGYNLADEGGNGDDLGNGD<br>GGSGGGDGDADDDGDGSGYGGGAGGDDGNDYEYNDYYSDG | 323.79 | 176.27 | -8.9231 |
| **protein6** | DDDDDDNDDGGGEGGNDDGGEEDDRDGGGNYGGGGGMDDGGN<br>GANGYDGGSNLANGGGGLNSYAGAPPASNGYRGNNYGNYVNYG<br>RNGSYAYNRYSYSRFGNNSARANNGGGGNSRGGSRNRAGHGRGR<br>GRGRGQGNRGNRGSRGGRGGRSRRRGRGRGGGGQSGGRD | 312.42 | 177.14 | -8.9920 |
| **protein7** | DGDGQDGDGDEGEGGGDGGDNDGGYNNGGEGGGGGNDNDDSG<br>GGNNGSAMSADGGVGGANSYNYRNSRDGYNYGNGAYFRAYPAY<br>GRDYNYRLANRNNRNYGARGPLGNDGSHNNSSGRGNGGGGGNG<br>AGGGGRGSGNGRRRSGQGGRRRRGRSRRRGDSRGRGGRGD | 328.67 | 166.73 | -8.6177 |
| **protein8** | FLLYPYYSGSSNGDNYDANADADAAGDGDNEEGDESDDNDRSDS<br>GDMGYNGGGGNGNDGGGDGGGGNGRRGGGDGGGGGDNGGGR<br>GGGGGRGGQQNNRRGRGGGGGRNRRNARDRNRGGNGRRGAGR<br>NGGGRGRRRRASGRSHGSNSGAGNRYGNYNPGNYYNSYVSY | 317.78 | 170.41 | -8.7060 |
| **protein9** | RYEGGSYRYRGSGGRRNRRRNSRGLRGDRGGRRRGGGGGRGGS<br>RYRGRGRRNGHGNRSNGGGDNGYGSGGRDGNYGGSNFYNNNAA<br>GSGGNEGGGRADPYGGGNNGGGAVDALYNSGGNNGDAGGANAS<br>DGGDGADDGGRDYQDPEDQDGMDDGDGYGNNGSNNSGNG | 312.49 | 168.68 | -8.7439 |
| **protein10** | RNANGRNNAGGNNGGGYGRNGASPQGGNSDARGGGLDGYGRRR<br>GRRRHRQNRYARAGNSGDGNGRGDGRGGYRSRRSNGDGGGGGG<br>GGRGNNSSGGGPYMGNGDGGGNGDRGGGRAGNDSGDARSGSLG<br>GGGRGNDEVSYNDGNFGGGNDYDYDDDRYEDSYNNAEYG | 311.84 | 165.43 | -8.4160 |
| **protein11** | DDGGDGDRRGGDEGGDGNDGDDGGGSDNGGGGNNSGGGNNGG<br>GDGNANSGAGAGNGRVAGNANNSYSYNGNYPSPMGSYRGAYSDN<br>FSNNYHLSYRRNRRYLGRANARGYNGGSRYSGRRAQYRGGRNGG<br>GGNGGRGRRGRGGGGQGGGDGRNERGGGDRGEGRDDDR | 302.85 | 170.91 | -8.2059 |
| **protein12** | YYYSYAYRHSANYRNRSNGRNNPGRRAGSRRRNRSGANGGGGGG<br>GRGRRRNGRDGQRRGGGGRGGGNGGARGGDDNDAGDDGEDGD<br>GGGGDEGGGNDDGSGDRELDGGDGDGNGNDGGGSGGGGNGAR<br>NGGSNGPNNNGSSGSRADRNGNQSVGGYNGMFLAYRNYYY | 312.05 | 180.99 | -8.3050 |
| **protein13** | PYGNHMGNAYYGYGYGYQPNGGSGGSGNSAGSNNAGYAGGGRG<br>NRRDGGNNERGGDGRDGGNDRDRSDGRRGDGRDGGAEGRGRG<br>GGDRNGNGGGDERDRNDDDRSRGGDGNGDNRAGGNRRRGGQG<br>DNSGGRGSGGARSGSGGRGSNAGGFNGNNYGLLNVAYNSYY | 277.46 | 155.97 | -4.0120 |
| **protein14** | ARSSGDNLNGGDGRSARNGAGRNGGGGGYGGRSDGGPRDARYS<br>SGVNGRNGLYGFPGDYGRDGGYDNGNNNAGRGGSDNRGRGGNG<br>GYGGGRAGGGGSENNRDRGHDGYRGGQGGNGNGGANYSRQDR<br>GDGNASNDEGRDGNRGDGGRGSMESNNYRGDRRGGYYAND | 274.37 | 162.22 | -4.4784 |
| **protein15** | RRGEDQERDGDRGDGGRGGGRGNRGGGSGGGSGGNGGSRAGGS<br>GDGNYNAGSGNGGGSMNSSGNGNGYHAYGVNYLAPYRYYPNFR<br>DNNYGGYYNAGGNSYGLANNSANSANSNQGRGNGAGGNGDRGG<br>GGGGGNDGNDRGRRDRGGGGGRGRRRDGRRDRRDDDDDE | 283.52 | 168.23 | -5.2371 |

# References

[1] Urry DW, Gowda DC, Parker TM, Luan CH, Reid MC, Harris CM, et al. Hydrophobicity scale for proteins based on inverse temperature transitions. Biopolymers: Original Research on Biomolecules. 1992;32(9):1243–1250.
